# Digital Health and Inequalities in Access to Health Services in Bangladesh: Mixed Methods Study

**DOI:** 10.2196/16473

**Published:** 2020-07-21

**Authors:** Tanvir Ahmed, Syed Jafar Raza Rizvi, Sabrina Rasheed, Mohammad Iqbal, Abbas Bhuiya, Hilary Standing, Gerald Bloom, Linda Waldman

**Affiliations:** 1 Institute of Development Studies Brighton United Kingdom; 2 Department of Oncology and Medicine Faculty of Medicine, Dentistry and Health University of Sheffield Sheffield United Kingdom; 3 Johns Hopkins University Dhaka Bangladesh; 4 International Centre for Diarrhoeal Disease Research, Bangladesh Dhaka Bangladesh; 5 Independent Public Health Expert Dhaka Bangladesh

**Keywords:** health equity, eHealth, mHealth, digital health, health technology, Bangladesh, developing countries

## Abstract

**Background:**

Globally, the rapid growth of technology and its use as a development solution has generated much interest in digital health. In line with global trends, Bangladesh is also integrating technology into its health system to address disparities. Strong political endorsement and uptake of digital platforms by the government has influenced the rapid proliferation of such initiatives in the country. This paper aims to examine the implications of digital health on access to health care in Bangladesh, considering who uses electronic devices to access health information and services and why.

**Objective:**

This study aims to understand how access to health care and related information through electronic means (digital health) is affected by sociodemographic determinants (ie, age, gender, education, socioeconomic status, and personal and household ownership of mobile phones) in a semiurban community in Bangladesh.

**Methods:**

A cross-sectional survey of 854 households (between October 2013 and February 2014) and 20 focus group discussions (between February 2017 and March 2017) were conducted to understand (1) who owns electronic devices; (2) who, among the owners, uses these to access health information and services and why; (3) the awareness of electronic sources of health information; and (4) the role of intermediaries (family members or peers who helped to look for health information using electronic devices).

**Results:**

A total of 90.3% (771/854) of households (471/854, 55.2% of respondents) owned electronic devices, mostly mobile phones. Among these, 7.2% (34/471) used them to access health information or services. Middle-aged (35-54 years), female, less (or not) educated, and poorer people used these devices the least (α=.05, α is the level of significance). The lack of awareness, discomfort, differences with regular care-seeking habits, lack of understanding and skills, and proximity to a health facility were the main reasons for not using devices to access digital health.

**Conclusions:**

Although influenced by sociodemographic traits, access to digital health is not merely related to device ownership and technical skill. Rather, it is a combination of general health literacy, phone ownership, material resources, and technical skill as well as social recognition of health needs and inequity. This study’s findings should serve as a basis for better integrating technology within the health system and ensuring equitable access to health care.

## Introduction

### Background

**Figure 1:**
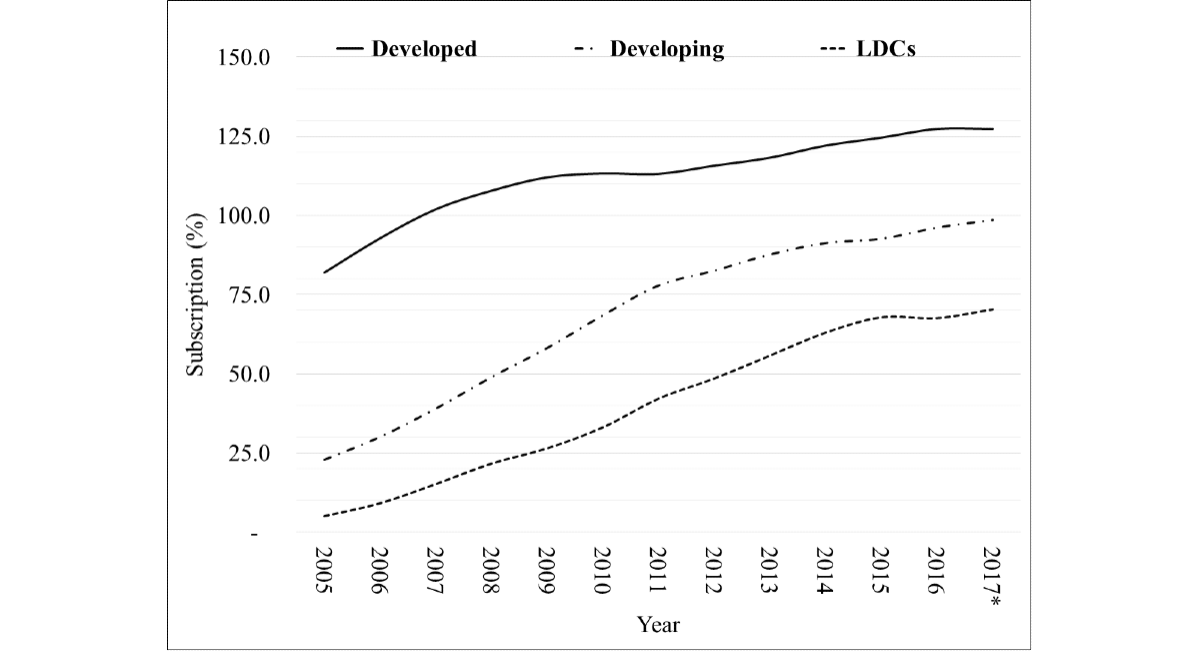
Mobile-cellular subscription per 100 people by developed, developing, and least developed countries. LDC: least developed country.

Globally, there has been an impressive growth in the number of cell phones and internet users, and the price of services and devices has decreased [1,2]. Mobile phones have become a thriving market, characterized by 7.7 billion (estimated) subscribers in 2017. Globally, the proportion of the population covered by at least a second generation (2G) network grew from 58% in 2001 to 95% in 2015. Internet penetration grew from 6.5% in 2000 to 43% in 2015, and the proportion of households with internet access at home increased from 18% in 2005 to 46% in 2015 [3,4]. The mobile-cellular subscription per 100 inhabitants has exceeded 100% in developed countries, and both developing and least developed countries (LDCs) are racing toward similar levels. Since 2005, most subscriptions have come from developing countries and LDCs, and the gap between these and developed countries is reducing (Figure 1) [3].

According to the International Telecommunication Union, a 10% increase in internet speed can increase economic growth by 1.3% in low- and middle-income countries (LMICs) [5,6]. Given such growth and potential, both national and personal spheres are now influenced by digital innovations, as governments strategize to expand coverage of developmental initiatives by reaching remote areas or individuals who engage in web-based shopping or personal banking. In 2014, the mobile-cellular industry generated US $3 trillion, which contributed to 3.8% of the global gross domestic product, and this number is expected to rise to 4.2% by 2020 [7]. This economic contribution came about largely by linking previously unconnected communities, financial inclusion through eCommerce (use of electronic means including mobile phones for financial purposes such as transferring money, everyday banking etc), and designing and delivering innovative solutions for improving quality of life (eg, sharing information, providing services, technologies for mass production). Examples include Tigo Kilimo by Tigo in Tanzania (launched in 2013) [7] and Airtel Green SIM in India (launched in 2007) for eAgriculture (use of electronic means including mobile phones to provide and access information regarding farming techniques and related products and market) [8,9] and TradeNet in Ghana [10] and bKash in Bangladesh [11,12] for eCommerce and mCommerce (using technology including mobile phones for financial transactions) as mobile wallets. M-Pesa by Vodafone is one of the largest mobile-based financial services in the world, used by millions across Africa, Europe, and Asia [13-15]. The information and communications technology (ICT) industry is now a popular area for employment worldwide. In 2014, the mobile industry directly employed approximately 13 million people and indirectly supported 12 million more [7,16-18]. Similar to other development domains, health is also exploring the potential of technology in improving the well-being of people, including health-related well-being, which can range from improvement in health ailments to acquiring the necessary information about health conditions or services.

The integration of technology within health began as electronic health (eHealth), the use of electronic platforms for the provision of health information and services, data collection and management, etc. When mobile phones are used to do the same, it is called mobile health (mHealth) [19]. Currently, because of the advent of artificial intelligence (AI) and big data, eHealth and mHealth are now called digital health. Although digital health indicates a much broader and smarter technology horizon, eHealth and mHealth remain the major forms of technology that are being endorsed for system integration to improve health system performance and increase peoples’ access to health care [20]. Therefore, there is a growing number of conferences, workshops, websites, apps, and publications regarding how electronic platforms can be implemented and integrated within the health domain [1,2,21-23]. Bangladesh is also in the process of doing the same. Currently, the country has 4 mobile cellular operators and a significant subscription base (149 million). Evidence suggests that a large proportion of households own mobile phones (81%) [24-28]. The government’s commitment to digital development, popularly known as Digital Bangladesh, has helped in fostering strategic and policy direction to adopt ICT in health care (and other development domains) [29-32]. Due to the large subscription base and strong political mandate, there are ≥42 internet-, SMS text messaging–, and call center–based eHealth and mHealth initiatives in the country providing awareness regarding maternal health, drug and alcohol abuse, smoking cessation, HIV/AIDS, and general health care [19,29,33-36]. Despite these initiatives, there is little systematic evidence of the impact of technology on equitable access to health services and information, especially in resource-poor settings such as Bangladesh.

Access to health includes availability, geographic location, affordability, and acceptability (by the community) of services. Considering these dimensions, the challenge is to ensure that everyone, irrespective of their social group, gender, etc, can access the required health care [37]. Bangladesh, despite its successes, has several health system challenges and prevailing health disparities, resulting in limited access to and utilization of quality health care [38,39]. Globally, one of the assumptions of the integration of technology for health care (and other development initiatives) is that digital health can contribute to equitable access to health care, especially in LMICs. However, there is evidence that access to electronic platforms can be hindered because of sociodemographic, gender, and geographic barriers—a state of disparity called the digital divide [40,41]. This divide bears great importance, especially when technology is being endorsed for system integration to improve access to health that can ensure universal health coverage (UHC) in Bangladesh [42]. Considering the growth of ICTs and the proliferation of digital health initiatives, a framework that defines the dimensions of access to health information and services through electronic means can contribute and is crucial to this integration. This is because the rapid growth of ICTs and related development solutions can often be misinterpreted as being equivalent to access and use.

### Objective

This study aims to understand the degree to which ICTs enable Bangladeshi people to increase their access to information and improve their well-being by exploring the factors that influence whether people have access to, and use, digital health information and services (this refers to non-AI–, machine learning–, or big data–based initiatives, previously called eHealth and mHealth). It focuses on who owns electronic devices and uses their device to access health services and/or information and the implications of this for health equity. The findings can be useful in devising a framework to ensure equitable access to digital health information and services in Bangladesh and relevant contexts.

## Methods

### Study Setting, Population and Data Collection Tools and Techniques

The data presented in this paper were collected using a mixed methods design. The quantitative data, supported by research funding from the UK Economic and Social Research Council, comes from a household survey to understand the role of ICTs in health information seeking conducted between October 2013 and February 2014 in a subdistrict of Bangladesh called Mirzapur in the Tangail district. Mirzapur was chosen because it is semiurban with typical sociodemographic characteristics of other such periurban subdistricts in the country [43]. As there were no previous variance estimates of outcome variables, 0.5 was used to calculate the required sample size to obtain 95% CIs with plus or minus 10% precision and a design effect of 2. Thus, a sample size of 2401 was obtained, which finally became 2527, considering a 5% nonresponse rate. This was calculated for 3 settings: urban (Dhaka), rural (Chakaria), and semiurban (Mirzapur). Therefore, each setting ended up with a subsample of 842. In Mirzapur, the sample size reached 854 households. Sample households were selected from 28 villages (30 households per village) using systematic cluster sampling from a Health and Demographic Surveillance System (HDSS) [44]. Approximately 81% of the information was gathered from the household head or the spouse of the head. Where neither the head nor the spouse was present or able to take part, an adult child of the head or the child’s spouse was interviewed. The survey questionnaire was developed and pretested both in Bangla and English (the Bangla version was used to collect actual data) on a browser-based, open-source platform using locally available 7-inch tablets with Android version 4.0. The use of digital means to seek health information or services was explained to the respondents as if the respondent (and/or any member of the household) had heard of or used phones or computers to seek information about any health issues or services via voice call, SMS text messaging, or internet browsing.

To further unpack the survey patterns and findings, 20 focus group discussions (FGDs) were conducted from February to March 2017 as part of the first author’s PhD fieldwork. Respondents, who had never used electronic devices to seek health services or information, were selected from 6 FGD groups constructed from the HDSS using typical demographic and economic traits: 8 rich and poor young female and male (college students) groups, 8 rich and poor middle-aged female and male groups, and 4 rich and poor elderly female and male groups.

### Data Analysis

The principal component analysis technique was used to elicit asset scores, and its distribution was arranged in quintiles to create the income groups. The lowest 2 quintiles were grouped as poor, and the highest 2 quintiles were grouped as rich. The distribution of the middle quintile was halved, and the lower half added to the poor group and the upper half to the rich group to remove the complexity of constructing the FGD groups. As it was a qualitative exercise, further discussion was conducted with the group during the FGDs to subjectively triangulate the accuracy and related homogeneity of the participants' income status. Each group consisted of 4 to 5 participants, and the discussions took place at locations preferred by the participants, that is, households (for female respondents) and local gathering places (for men). Each session lasted approximately 30 min, facilitated by the first author (male and a PhD student). During discussions, although agreements in opinions were sought, disagreements were also noted along with the respondents’ body language. A female notetaker was present during discussions of the female groups. Discussions were conducted and later transcribed in Bangla and then into English for internal consistency.

The sociodemographic analysis was conducted by identifying the frequencies of the respective characteristics/traits. Information on socioeconomic status (SES) was collected from the HDSS as asset scores, a popular method called the asset index. The range of scores was divided into 3 equal categories (lowest as poor, middle, and highest as rich), and frequencies of a sample belonging to the respective categories were identified. The ownership of electronic devices and the use of devices were analyzed for (1) seeking information; (2) health services or information; and (3) demographic factors such as age, gender, education, and income to stratify the results. A quantitative analysis, including tests of significant, was performed using STATA version 14 (made by StataCorp), and Microsoft Excel was used to construct graphs and some amount of data management. The patterns indicated by the quantitative analysis were further explored qualitatively using content analysis techniques [45,46]. Altogether, 3 broad themes emerged: (1) reasons for not using mobile phones or other electronic devices, (2) the extent of awareness of electronic sources of health information, and (3) the role of intermediaries (family members or peers who helped to look for health information using electronic devices). These were analyzed by reference to the gender, SES, and education of respondents. Both analyses were part of PhD studies supported by the International Development Research Centre.

### Ethical Consideration

Ethical approval was obtained from the institutional review boards of the survey partners: International Centre for Diarrhoeal Disease Research, Bangladesh, and Institute of Development Studies, University of Sussex (for qualitative data collection). Before data collection, the study was explained to the participants, and written consent or thumbprints were obtained. During data collection (both qualitative and quantitative), informed consent was obtained from all participants.

## Results

### Sociemographic Profile of the Respondents and Ownership of Electronic Devices

Tables 1-3 show the sociodemographic characteristics and ownership of electronic devices of the survey households and respondents. The mean age of the respondents was 41.3 (SD 14.5) years, and 67.2% (574/854) of the respondents were between the ages of 25 and 54 years. A total of 38.3% (327/854) had less than primary education, and a small proportion (26/854, 3.0%) were graduates and above. More than half (494/854, 57.9%) of the households had 4 to 6 members, representing the usual Bangladeshi family size [47]. In all, 71.9% (614/854) of the respondents were female. There were more female respondents because the survey was conducted during the daytime when male members were away. A total of 73.1% (625/854) of the respondents were not employed (being a housewife is not considered a job in Bangladesh), which was not intentional. Conversely, 77.3% (660/854) of the household heads were employed. A total of 95.9% (819/854) of households had a regular income, and 92.6% (791/854) of households had no social security card (in Bangladesh, having a social security card indicates that the corresponding household belongs to the extremely poor socioeconomic group), a common feature of semiurban Bangladesh. In all, 55.2% (471/854) of respondents had personal electronic devices and 90.3% (771/854) of the households owned at least one, which was almost always a mobile phone. The personal ownership was considerably low, perhaps because the survey population had male:female ratio of 1:3 and in a context like Bangladesh, women have less access (in this case ownership) to devices compared with men. However, in the case of household device ownership, family members tend to share electronic devices, and there was always a chance of a lack or less use of devices due to the influence of other family members. Therefore, to understand the equity implications of access to electronic devices, although lower, personal ownership was considered in this study rather than shared ownership.

**Table 1:** Demographic statistics of the respondent (N=854).

Parameter		Values
**Age (years), mean (SD)**		41.3 (14.5)
	16-24, n (%)	97 (11.4)
	25-34, n (%)	215 (25.2)
	35-44, n (%)	190 (22.3)
	45-54, n (%)	169 (19.8)
	55-64, n (%)	110 (12.9)
	>65, n (%)	73 (8.6)
**Gender, n (%)**		
	Male	240 (28.1)
	Female	614 (71.9)
**Education, n (%)**		
	Less than primary	327 (38.3)
	Primary	206 (24.1)
	Secondary	254 (29.7)
	Higher secondary	41 (4.8)
	Graduation and above	26 (3.0)
**Respondent employment status, n (%)**		
	Yes	229 (26.9)
	No	625 (73.1)

**Table 2:** Household-level information of the respondents (N=854).

Parameter		Values, n (%)	
**Members per household**			
	1-3		257 (30.1)
	4-6		494 (57.9)
	>7		103 (12.1)
**Household head working status**			
	Yes		660 (77.3)
	No		194 (22.7)
**Socioeconomic status of the household**			
	Poor		295 (34.5)
	Middle		276 (32.3)
	Rich		283 (33.1)
**Presence of menial labor^a^**			
	Yes		35 (4.1)
	No		819 (95.9)
**Household’s social security card**			
	Yes		61 (7.1)
	No		791 (92.6)
	Don’t know		2 (0.2)

^a^Refers to jobs such as housemaid and unskilled day laborer.

**Table 3:** Electronic device ownership of the respondents (N=854).

Parameter		Household, n (%)	Personal, n (%)
**Ownership of devices**			
	Total	771 (90.28)	471 (55.2)
	Mobile	751 (87.9)	454 (53.2)
	Laptop	2 (0.2)	0 (0.0)
	Both	18 (2.1)	17 (2.0)

### Access to Health-Related Information or Services by Respondents Who Owned Electronic Devices and Influence of Sociodemographic Characteristics

Tables 4 and 5 show that everyone who owned mobile phones or laptops/computers had used those to communicate or seek information, which included day-to-day conversations to seeking specific services, such as agriculture or other government-related information. It also showed that the predominant means of seeking information was voice calls, followed by SMS text messaging. Analysis of health information seeking for any health concern, including information only, or services showed that of all people who sought health information, a number (22/34, 65%) reported seeking information/services for minor health issues. Although everyone had used their device to seek some form of information, only 7.2% (34/471) sought health services or health information.

The use of electronic devices by the people who owned personal devices was stratified by sociodemographic characteristics of age (*P*=.02), sex (*P*=.41), education (*P*=.004), and SES (*P*<.01; Table 6). Although overall use was low, the pattern suggests that among those who used their devices, people who were middle-aged (35 to 54 years), women, and poorer people who had less or no education used them less than others. The difference in use was found to be significant on the chi-square test.

**Table 4:** Percentage of use and awareness of the use of devices to communicate and seek information by personal device owners (cell phones, laptops, or both; n=471).

Parameter^a^			Used and aware, n (%)		Not used nor aware, n (%)
**Any information**					
	Total	471 (100.0)		0 (0.0)	
	Voice call	471 (100.0)		0 (0.0)	
	SMS text messaging	226 (48.0)		245 (52.0)	
	Internet	26 (6.0)		445 (94.5)	
Health-related information			34 (7.0)		437 (92.8)

^a^Multiple responses.

**Table 5:** Percentage of communication device use to seek health information (n=34).

Parameter	Values, n (%)
Minor health issues	22 (64.7)
Serious health issues	12 (35.3)

**Table 6:** Percentage of personal device owners who sought health information or services by age, sex, education, and socioeconomic status in Mirzapur (n=471).

Parameter		Used and aware, n (%)	Not used nor aware, n (%)	*P* value
**Age in year^a^**				.02
	Young adult	5 (8.2)	56 (91.8)	
	Adult	15 (9.9)	137 (90.1)	
	Middle age	5 (2.8)	175 (97.2)	
	Elderly and above	9 (11.5)	69 (88.5)	
**Gender**				.41
	Male	13 (7.9)	151 (92.1)	
	Female	21 (6.8)	286 (93.2)	
**Education^a^**				.004
	No education	5 (4)	120 (96)	
	Primary	4 (3.4)	113 (96.6)	
	Secondary	14 (8.4)	153 (91.6)	
	Higher secondary and above	11 (17.7)	51 (82.3)	
**Socioeconomic status^a^**				<.001
	Poor	2 (1.4)	137 (98.6)	
	Middle	8 (6)	126 (94)	
	Rich	24 (12.1)	174 (87.9)	

^a^Statistically significant (*P* value is less than .05).

### Barriers to Accessing Health Information and/or Services by the Owners of Electronic Devices

People who did not use electronic means to access health care or information were further interviewed over FGDs to explore their reasons. Almost everyone has accessed some form of electronic information at some point using mobile phones, mostly through voice calls to an office (ie, local agriculture office or bank) to ask for information. However, despite ample promotion and publicity, the provision of electronic health information or services and the words digital health were unfamiliar to many. One male student mentioned:

I have never heard the word digital health until today. No one told us that one can get health-related information in this way. But sometimes we make calls to some office to know about things. In this way we can get information easily.

Female students had slightly different views. They preferred to call their friends and/or family for information, as one explained:

We use mobile phones to talk about many things. If we need to know about something, we call our friends or elders. But I can’t remember if we have ever talked about digital health.

Nonetheless, some of them also use mobile phones to seek health-related information despite a lack of familiarity with formal words such as digital health. Most of the participants had asked for advice from a family member or from someone who had relevant knowledge. As a result, most were reluctant to use electronic means, and only a few had used their device to seek health information, such as the internet and call center. This indicates that people use their phones to ask advice from friends, family, or social acquaintances but are not aware of or do not use formal digital health services. As one of the middle-aged female participants said:

We are ignorant people, we don’t understand all these. Besides we don’t need this [digital health], it’s enough if you can receive and make a call using a phone.

The reasons mentioned in the FGDs for not using electronic means to seek health information and/or services can be summarized into 4 major reasons.

#### Awareness of Digital Health Services

Many participants were not aware of eHealth services. This was usual in Bangladesh, given the ongoing promotion of the telecom industry in Bangladesh. A few younger respondents could mention GrameenPhone’s health helpline (789), but most of them had a general lack of understanding of eHealth services. Respondents knew that 789 is a service to call physicians using GrameenPhone mobile phones but did not know how it worked. Other than lack of skill to navigate the device and the platform, lack of awareness regarding associated costs and how to talk about personal health ailments was a major concern for the participants.

Most of the participants were confused by the promotional activities undertaken by telecom companies. Mirzapur has an abundance of kiosks and shops that offer a range of mobile phone–related services and products with colorful banners and posters displaying information about these services. Considering the overflow of information on display, FGD participants described it as “difficult to distil information related to digital health services.” One of the male participants said:

The local shops are full of pictures and words about hundreds of offers. Among those, I don’t remember any explaining the availability of such type of health service [digital health]. If we can’t find one, how can we be aware that such services exist?

The young and educated participants were more aware of digital health services compared with others. However, even among this group, most did not know much about these services. Some of them were aware of social media–based health initiatives and promotions. Almost everyone had a Facebook account and had seen adverts and information related to health. Although social media (including Facebook) mostly presented information on diet, healthy lifestyles, and beauty tips, some serious issues such as cancer, HIV, and AIDS have also been presented. As one of the female students explained:

We don’t know what it is [eHealth] and how can we get health information through it. Or how does it work, how much money it takes etc. Most of us use Facebook or at least have seen it. I sometimes get posts related to beauty or diet-related tips. Sometimes I get information on cancer. But I don’t know what eHealth is. I think if digital health services can be made as easy as Facebook, then everyone will come to know about it.

#### Personal Comfort and Acceptance

During FGDs, respondents (mostly women) expressed specific concerns regarding not knowing the counselor/provider personally and were hesitant to talk about personal issues and illnesses. One female respondent said:

We are rural ignorant people. How can we talk about illnesses to someone, whom we don’t know or see? We are shy and just can’t do it.

Middle-aged respondents expressed their lack of trust in the accuracy and quality of digital health information and preferred face-to-face interactions with the person providing the information and advice. Almost everyone preferred to discuss health care needs with their friends and family first, then with local drug sellers and village doctors (quacks) before taking them to formal medical providers. If someone advised them, only then did they consider seeking health information or service electronically. Young and educated respondents were more inclined to use eHealth as they perceived it as ensuring one’s confidentiality and privacy. However, they had concerns about cost. A young male participant said:

It takes up money from mobile account balance. Both internet and talk time are expensive. But it’s true that you can say many things over a phone which is rather difficult in a face to face consultation with a person whom you know.

#### Literacy and Skills

Some participants mentioned that they lacked the skills needed to access digital health effectively. This ranged from proficiency in and with English (and Roman letters) and technical ability to navigate the device and its software (ie, specific app, browser, and internet settings). For example, calling a call center entails the ability to press specific numbers in response to questions or directions. Alternatively, browsing the internet requires English literacy and technical skills to set their devices for internet use. One of the middle-aged participants said:

It’s easier for young people. They know how to do this using their mobile phones or computers. They also have the skills to do that. That’s why I don’t have internet in my phone.

Some young participants also felt that lack of proficiency in English is a barrier to accessing information and services electronically. One of them mentioned:

We are Bengali people and Bangla is our language. We are not very good at English which, in my opinion, is our main problem

Some of the young participants mentioned that family members had asked them (or their friends) to look for health information electronically, but they never looked for it themselves. One of the elderly respondents said:

We are old and that’s why we don’t know much about it. We can only receive and make calls. Sometimes if someone sends an SMS, we take the phone to the other members of the house to find out what it is. We do the same when we want to know something about the phone.

#### Proximity to Health Facilities

One of the reasons why respondents did not engage with digital health in Mirzapur was the availability of and access to conventional, formal health services within their vicinity. Mirzapur has a public hospital, the Upazila (subdistrict) Health Complex, and a philanthropic hospital (Kumudini). For any medical emergency, anyone can visit these hospitals instead of using a call center or other digital health services. During discussions, participants agreed that it could be one of the reasons for their reluctance to use digital health services, including information. One of the middle-aged participants mentioned:

Kumudini hospital was established long time ago and is just beside our house. It is much easier and more comfortable for us to visit this hospital when needed. Besides we also have the upazila hospital.

## Discussion

### Principal Findings

This study aims to examine the current linear understanding that access to technology results in access to health care by all. Considering digital health services as a proxy for the integration and implementation of technology for providing health care and information, it has used sociodemographic characteristics to explore which population groups have access to technology and have accessed digital health services. In more elaborate terms, it focuses on the degree to which ICT and technology has enabled Bangladeshi people to increase their access to information and improve their well-being by exploring the factors that influence whether people have access to electronic devices (namely, mobile phones or personal computers/laptops) and use these devices to access health services and/or information digitally. The findings show that although there is high household ownership (771/854, 90.3%) of mobile phones, personal ownership is much lower (471/854, 55.2%). Everyone who owned a personal mobile phone used it to seek information and services electronically, but only a small proportion (34/471, 7.2%) used it to seek health-related information or services. Although the data suggested younger men and those with a higher education and SES chose to use their devices to access digital health, there was little statistical evidence that sociodemographic factors influenced the use of digital health information and services. According to the findings from the FGDs, nonuse of devices to seek health-related information or services was related to the perception of digital health as an unfamiliar health care–seeking model. Other factors were lack of technological skills and related literacy to seek electronic information or services, associated cost of accessing information, lack of awareness about digital health services, and proximity to functioning health centers.

In the context of rural Bangladesh, previous works have reported slightly lower household ownership of mobile phones, but with an upward trend [28,31,48]. The data reported in this paper were collected later, and in a semiurban context (Mirzapur), which is adjacent to Dhaka. Mirzapur is thus likely to have greater access to technology and resources than rural Bangladesh. The high ownership of electronic devices found in this paper is consistent with what others have reported. However, if ownership of devices is used as a proxy for access to digital technology, the data show that only about half of the respondents have personal devices. As there is a dearth of evidence regarding the personal ownership of devices in Bangladesh, it was not possible to compare the findings with the situation in other parts of the country. Nevertheless, the general idea that high household ownership and subscription to mobile-cellular networks means high access to technology needs to be reconsidered and explored further.

### Socio-Demography of the Use of Digital Health

Although this paper has shown that only a small proportion (7.2%) of owners have used their phones to access digital health for health services or information, in the rural context, use of devices to access digital health has been reported to be even lower (2%) [28]. The difference in the spread of technology over time and context and related access (semiurban versus rural) can be the reason for this difference. However, such low use of devices generally to access digital health does not indicate that everyone is unable to access services or information digitally. In line with these findings, globally (including Bangladesh), male, young, educated, and wealthier groups are more likely to use their electronic devices to seek general information and health information or services [49-52]. A recently published paper, based on the findings from Mirzapur, reported that the use of mobile phones to access health information at least once in the last 12 months was 45% in college students (young and educated adults) compared with 18% in the general population. It also reported that internet users were predominantly (two-third) male phone owners [26]. Therefore, this paper strongly argues that any attempt to integrate technology in the health systems of Bangladesh (and similar contexts) and to endorse related digital health innovations must take into account sociodemographic attributes and the fact that services are more likely to be accessed by young, educated, and male populations. Although this represents a potential disparity in access to digital health, it also positions young and educated people to help the diffusion of technology in the community as change agents and therefore paves the way toward the much discussed and desired integration of technology into the health system to meet the challenges of UHC in Bangladesh and related contexts [42].

### Other Factors Influencing the Use of Digital Health

The other reasons for the low uptake of digital health include lack of awareness about digital health in terms of its modus operandi and availability, lack of personal comfort and acceptance of this form of health service or information, lack of literacy and skill for using digital health technology, and proximity to other health facilities that provide emergency care. However, these reasons are not mutually exclusive, and the relationship within and between them must be examined further in terms of underlying equity challenges. Awareness regarding digital health initiatives is possibly the first and foremost of these reasons, yet communicating the potential of digital health is not, as discussed above, straightforward. Many middle-aged residents have access to household resources and the relevant educational achievements (Table 1) that would make accessing digital health possible, but they are not sufficiently informed and do not have the technological skills (Table 2). They are simultaneously disinclined to pursue health services or information provided in this manner because they are unfamiliar with and do not trust the mode of delivery. Moreover, should they or their family members have a health need, particularly an urgent or emergency one, they would be able to access Mirzapur’s other health facilities. Young people, by contrast, are aware of the potential of digital health and have the relevant skills and literary sophistication required (Table 1). As indicated by others, they do not have the material resources and influence that would support and facilitate access to digital health (ie, financial resources and decision-making capacity in health care need) [26]. As a result, they tend to use this service when, as shown above, older people who have the necessary resources request that young people engage with digital health services or information. Young people, like the older generations, have access to Mirzapur health facilities when there is an emergency; however, their primary health concerns are, as others have reported and the earlier discussion indicates, private, nonurgent, and often deemed unnecessary, such that their concerns are dismissed and they are treated with disrespect [26].

The importance of sociodemographic and economic factors and related material factors was highlighted in other technology-based health interventions in Bangladesh. One of the popular mHealth interventions in Bangladesh called Aponjon, supported by the United States Agency for International Development and implemented in 2012, was designed to provide voice or text message–based pregnancy and newborn care. After studying the impact of the intervention, it was concluded that exposure to Aponjon messages was not associated with improved maternal and child health outcomes, such as the presence of skilled birth attendance at birth, breast-feeding practices, or facility delivery. Rather, higher sociodemographic and income status of the mother was more significantly associated with reported improvements [53]. Another intervention study was piloted to understand the feasibility and acceptability of a mobile phone-based intervention that combined counseling and the payment of cash transfers to improve the perception of both maternal nutrition during pregnancy and nutrition of the infant in rural Bangladesh. Although the study could not conclude the intervention as effective, it highlighted other material issues such as charging the mobile phone or spending cash (obtained from the project) to feed one’s family rather than focusing on the nutrition of the pregnant mother or infant as major barriers to the effectiveness of such mHealth interventions [54].

### Conclusions

There is high awareness and use of electronic devices to access electronic information and/or services across various sociodemographic categories in Bangladesh. Global trends suggest that access to the use of mobile phones and computers is expanding, including contexts like Bangladesh [15,31]. However, Mirzapur’s respondents continued to avoid digital health initiatives. Why? The only paper that has tried to explain the reason for such low use in Bangladesh concluded that, although the community has some technological readiness and will to use mHealth, lack of adequate human resources and technological abilities of the people may have restricted the use of electronic devices for health services or information [55].

Inequitable access to health care is one of the major health system concerns for LMICs, including Bangladesh. Expansion of coverage of health care provision and implementing efficient policy making and governance activities is critical for improving population access to health care. At the same time, adopting an equity focus can ensure the inclusion of various population groups. The central question in this paper is, therefore, does high access to technology mean that various sociodemographic groups can access digital health services, thereby having higher access to health care and information? The answer lies in how technology interfaces with other social determinants of health to produce equity and inequity. Shankardass et al [56] argued that health inequities are caused by complex social, economic, and political factors (ie, the influence of gatekeepers, affordability of services, provision of quality health care, and strategies to secure access of poor and vulnerable groups to health). These factors limit recognition of the need for and creation of proequity policies. Digital health promises to address access to health services and information [57], and, in demonstrating substantial growth in technological access, it gives the impression that challenges in access to health services and information are being addressed, leading to a decrease in the digital divide. However, as shown in this paper, this approach of ubiquitous access to technology obscures how inequity in access to health plays out. As Embrett and Randall [58] argue, addressing health inequity is dependent on generating public awareness to develop sufficient political incentives for change. However, the lack of access to health services and health information that, for instance, young people experience is not socially acknowledged. Digital health information and services offer some potential to address this challenge, with young people having appropriate awareness, sufficient skills, education, and literacy that would make this an attractive option, but they lack the English sophistication required to articulate health needs and use, and they do not have the necessary resources to turn this into a reality. Addressing inequity in digital health requires action to increase not only device ownership and the technical skills necessary to operate these devices but also the material resources to encourage their use and social recognition of health needs and inequity.

This paper presents evidence on the implications of the adoption of digital health services on equitable access to health. To capitalize on the growth of technology, it is important to recognize that without appropriate recognition, digital health services may result in underused services and can influence further disparities such as information-rich poor groups. This means that without the required approach, whereas some social groups will have more access to health care because of their better health, technology, and general literacy, others may become vulnerable and marginalized with restricted access. This should serve as a basis for any future attempt in devising (and adopting) operational frameworks that envision both accountability and equity for effective integration of ICT platforms (digital health) to address disparities and related health systems challenges for Bangladesh and other LMICs.

### Limitations of the Study

This survey was undertaken with the expectation of high levels of digital health usage. As a result, insufficient attention was paid to the use of mobile phones for health information through casual conversations among peers and kin.

## Abbreviations

AI: artificial intelligence
FGD: focus group discussion
HDSS: Health and Demographic Surveillance System
ICT: information and communications technology
LDC: least developed country
LMIC: low- and middle-income country
SES: socioeconomic status
UHC: universal health coverage
